# Genomic Binding Profiling of the Fission Yeast Stress-Activated MAPK Sty1 and the bZIP Transcriptional Activator Atf1 in Response to H_2_O_2_

**DOI:** 10.1371/journal.pone.0011620

**Published:** 2010-07-16

**Authors:** Majid Eshaghi, Jong Hoon Lee, Lei Zhu, Suk Yean Poon, Juntao Li, Kwang-Hyun Cho, Zhaoqing Chu, R. Krishna M. Karuturi, Jianhua Liu

**Affiliations:** 1 Systems Biology, Genome Institute of Singapore, Singapore, Republic of Singapore; 2 Department of Bio and Brain Engineering, Korea Advanced Institute of Science and Technology (KAIST), Daejeon, Republic of Korea; 3 Computational and Mathematical Biology, Genome Institute of Singapore, Singapore, Republic of Singapore; 4 Department of Biochemistry, Yong Loo Lin School of Medicine, National University of Singapore, Singapore, Republic of Singapore; Universidade de Sao Paulo, Brazil

## Abstract

**Background:**

The evolutionally conserved MAPK Sty1 and bZIP transcriptional activator Atf1 are known to play a pivotal role in response to the reactive oxygen species in *S. pombe*. However, it is unclear whether all of the H_2_O_2_-induced genes are directly regulated by the Sty1-Atf1 pathway and involved in growth fitness under H_2_O_2_-induced stress conditions.

**Methodology/Principal Findings:**

Here we present the study on ChIP-chip mapping of the genomic binding sites for Sty1, Atf1, and the Atf1's binding partner Pcr1; the genome-wide transcriptional profiling of the *atf1* 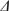 and *pcr1* 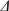 strains in response to H_2_O_2_; and the phenotypic assessment of ∼90 Atf1/Pcr1-bound or unbound genes for growth fitness under H_2_O_2_ conditions. ChIP-chip analysis shows that Atf1 and Pcr1 binding sites are overlapped in the genome and constitutively present before H_2_O_2_ stress. On the other hand, Sty1 recruitment primarily occurs at the Atf1/Pcr1 binding sites and is induced by H_2_O_2_. We found that Atf1/Pcr1 is clearly responsible for the high-level transcriptional response to H_2_O_2_. Furthermore, phenotypic assessment indicates that among the H_2_O_2_-induced genes, Atf1/Pcr1-bound genes exhibit a higher likelihood of functional requirement for growth fitness under the stress condition than the Atf1/Pcr1-unbound genes do. Notably, we found that the Atf1/Pcr1-bound genes regardless of their responsiveness to H_2_O_2_ show a high probability of requirement for growth fitness.

**Conclusion/Significance:**

Together, our analyses on global mapping of protein binding sites, genome-wide transcriptional profiling, and phenotypic assessment provide insight into mechanisms for global transcriptional regulation by the Sty1-Atf1 pathway in response to H_2_O_2_-induced reactive oxygen species.

## Introduction

Reactive oxygen species (ROS) are continuously produced as byproducts of aerobic metabolism. An excess level of ROS is highly toxic and has been implicated in the pathogenesis of several diseases, including cancer [1], [2]. Eukaryotic cells are thus equipped with various mechanisms to combat increased ROS levels [3]. On the other hand, ROS has also been found to be purposefully generated as signaling molecules to control various processes, including pathogen defense and programmed cell death [4], [5]. Therefore, maintenance of ROS homeostasis by sensing the level of ROS and controlling the defense mechanisms is critical for cell growth and survival. Hydrogen peroxide is a ROS-generating agent and commonly used in the laboratories to study the cellular response to (H_2_O_2_-induced) ROS.

In *Schizosaccharomyces pombe*, the mitogen-activated protein kinase (MAPK) Sty1 (aka Spc1 or Phh1) [6]–[9], homologous to the *Saccharomyces cerevisiae* Hog1 [10] and the mammalian c-Jun N-terminal kinase and p38 [11], plays a major role in response to ROS-generating agent H_2_O_2_ and many other stress factors. Upon stresses, Sty1 accumulates in the nucleus and plays a role in the activation of Atf1-dependent transcription and may be involved in mediating transcription through other basic-leucine zipper (bZIP) transcription factors such as Atf21, Atf31, Pap1, Pcr1, and Zip1 [12]–[17]. It has been shown that Sty1 is recruited to the promoter of *gpd1* and *hsp9*, the Atf1/Pcr1 target genes, upon osmotic, oxidative, and high temperature stresses [18], [19]. However, it is unclear if Sty1 is recruited to all Atf1/Pcr1 target genes in the genome. In this study, we address this question by using the ChIP-chip analysis for Sty1 binding sites on the genomic level.

Transcriptional profiling of fission yeast cells has shown that Sty1 regulates a large set of genes that are known as the core environmental stress response (CESR) genes [20], [21]. Majority of the CESR genes are also found to be dependent on the function of Atf1, a member of the ATF/CREB protein family and homologous to mammalian ATF-2 [12], [18], [22]–[27], indicating that Atf1 is a major transcription factor regulated by the MAPK Sty1 in response to H_2_O_2_ and a number of other environmental stresses. While Pap1 is found to be a redox sensor to low levels of H_2_O_2_ (e.g., ∼0.05mM H_2_O_2_) and could function independent of Sty1, Atf1 is a major player in response to high levels of H_2_O_2_-induced stress (e.g., ∼0.5mM H_2_O_2_) [28]–[30]. Zip1 has been shown to be specifically involved in response to arsenite and cadmium induced stresses in *S. pombe* [31], [32]. Disruption of *atf21* or *atf31* will lead to defects in mating, meiosis and sporulation, indicating their function in regulation of meiotic transcription [13], [14]. Atf1 and Pcr1 also play an indispensible role in regulation of meiotic specific transcriptions [14], [17]. In addition, Atf1 and Pcr1 are found to form heterodimer in vitro and in vivo [16], [22], [24], [27], [33]. Therefore, it has been proposed that the Atf1/Pcr1 heterodimer is important for their function. Nevertheless, *atf1* 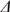 exhibits a severer phenotype upon oxidative stress than *pcr1* 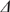, indicating that the Atf1/Pcr1 heterodimer cannot account for all of their functions in transcriptional regulation during response to H_2_O_2_ [18], [34]. It is possible that the heterodimer is essential for transcriptional regulation during meiosis.

Approximately one third of the induced and two thirds of the repressed CESR genes in *S. pombe* are found to share orthologs in the environmental stress response (ESR) or common environmental response (CER) genes in *S. cerevisiae* [20], [35], [36], indicating that the transcriptional response to environmental stresses is conserved in the two yeasts. Surprisingly, many ESR or CER genes in *S. cerevisiae* are found not to be required for growth fitness under the common environmental stresses [37]. Careful investigations of transcriptional profile of budding yeast under various growth conditions have demonstrated that many ESR or CER genes are actually associated with the growth rate, rather than the direct effects of stress [38]–[40]. However, it is unclear if fission yeast CESR genes are linked to the slow growth.

Physical binding at the promoter is a crucial evidence for a transcription factor to regulate transcription of a gene directly in yeast. Though the transcriptional response profiles of hundreds of CESR genes are found to be altered in *atf1* 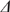 cells during response to a number of environmental stress factors, it is unclear if all of the Atf1-dependent CESR-genes are directly regulated by Atf1. We address this question though the analysis of Atf1 binding sites on a genomic scale using ChIP-chip technology. Because the disruption of *atf1* confers a severe phenotype under H_2_O_2_ stress condition, Atf1 target genes are likely to show a high likelihood of requirement for growth fitness under the stress conditions.

In this report, we show the study on ChIP-chip mapping of Atf1, Pcr1, and Sty1 binding sites at the genomic scale, transcriptional profiling of cells defective in Atf1 or Pcr1 function, and phenotypic assessment of ∼90 strains bearing a deletion allele of the Atf1/Pcr1-bound or unbound genes. Comparison analyses show that Atf1 and Pcr1 binding sites are overlapped and constitutively present before H_2_O_2_ stress. On the other hand, Sty1 binding is induced by H_2_O_2_ treatment and occurs primarily at the Atf1/Pcr1 binding sites. Among the Atf1/Pcr1-bound genes, some are found to be the H_2_O_2_-induced genes whose transcriptional response profiles are dependent on the function of Atf1 or Pcr1 and some are found be the non-responsive genes upon H_2_O_2_ treatment. Phenotypic assessment using the mini-culture growth curve assay shows that Atf1/Pcr1-bound genes regardless of their transcriptional responsiveness to H_2_O_2_ display a high likelihood of requirement for growth fitness under the oxidative stress conditions. Sty1 is found to be primarily recruited at the Atf1/Pcr1 binding sites after H_2_O_2_ treatment. Hence, our results provide insight into mechanisms for global transcriptional regulation by the Sty1-Atf1/Pcr1 pathway in response to H_2_O_2_-induced reactive oxygen species.

## Results

### Atf1 and Pcr1 binding sites in the genome are constitutively present before H_2_O_2_ stress

To determine the genomic binding profile of the bZIP factor Atf1, we performed the ChIP-chip analysis using a strain bearing the sole chromosomal copy of *atf1^+^-HA* allele. The *HA*-tagged strains showed no apparent growth defect under the H_2_O_2_ stress condition (see Figure S1 in File S1), indicating that the HA-tagged proteins are functional. Therefore, genomic binding profiles of Atf1 (Pcr1 or Sty1) was approximated by its functional HA-tagged protein in this study. We conducted the ChIP-chip experiments in HA-tagged cells prior to and after treatment with H_2_O_2_ at the final concentration of 0.5mM (see Materials and Methods). Independent repeats of ChIP-chip experiments were performed for reproducibility.

**Figure 1:**
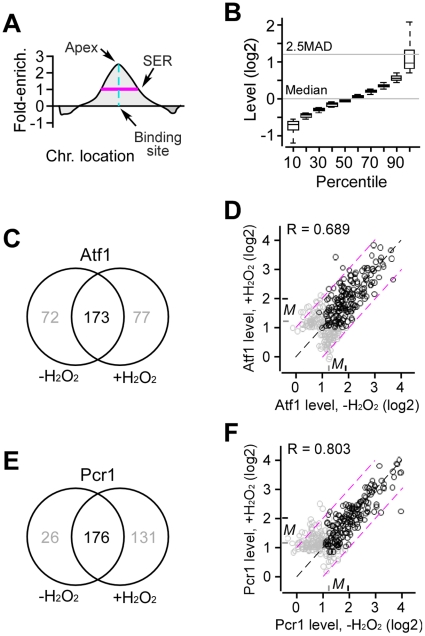
Atf1 and Pcr1 binding sites in the genome are present before and after H_2_O_2_ stress. (A) A schematic view of the protein occupancy in the genome-wide ChIP-chip analysis. Level of binding affinity is estimated by the ratio between the ChIP-enriched DNA signal and non-enriched (WCE) DNA signal or level of enrichment. Significantly enriched regions (SER) define the protein occupancy. Apex of the occupancy indicates the binding site in the chromosome. (B) Relationship between level of enrichment and rank of percentiles. The array median (Median) is set to 1 or 0 in log scale. Array features' signal above 2.5 MAD (or Median Absolute Deviation) plus the array median is used as a cutoff for significantly enriched signals. (C) Venn diagram showing the relationship between Atf1 binding sites found in cells prior to and after H_2_O_2_ treatment. (D) Scatter plot showing the relationship between the Atf1 binding levels found in cells prior to and after H_2_O_2_ treatment. Each dot indicates a binding site. Black dot and grey dot indicate the binding site found in both conditions and in one of the two conditions, respectively. *M* indicates the median level of the overlapping (black) and non-overlapping (grey) binding sites. Correlation coefficient (*R*) is indicated. Black dash line indicates the diagonal. Purple lines indicate boundaries within 2-fold change. All overlapping binding sites (black dots) display level change for less than 2-fold except for a few. (E) Venn diagram showing the relationship between Pcr1 binding sites found in cells prior to and after H_2_O_2_ treatment. (F) Scatter plot showing the relationship between the Pcr1 binding levels found in cells prior to and after H_2_O_2_ treatment. All overlapping binding sites (black dots) display level change for less than 2-fold except for a few.

Level of protein occupancies at various loci in the genome was indicated by the ratio between the signals resulted from hybridization with the chromatin-immunoprecipitated (ChIP) DNA (e.g., Cy5 labeled) and the whole-cell-extract (WCE) DNA (e.g., Cy3 labeled) (Figure 1A; see Figure S2 in File S1). All ChIP-chip microarray data were LOWESS normalized in which the array median signal level of all genomic features was set to 1 or 0 in log scale (see Materials and Methods). Occupancies were defined as those whose enrichment level was ≥2.5 *MAD* (*i.e.*, median absolute deviation) above the array median and were ranked within the top 3% (see Materials and Methods) (Figure 1B). The false discovery rate (FDR) of the identified binding sites in all ChIP-chip experiments was <4% (see Materials and Methods). The analysis identified 250 Atf1 binding sites that located at the probable promoter regions (that were defined as the upstream intergenic sequence up to 1Kb from the start codon [41] or 150bps from the TSS [42] (see Materials and Methods) in cells after H_2_O_2_ treatment. To test whether Atf1 binding sites in cells were present before H_2_O_2_ treatment, we determined Atf1 occupancies in cells prior to H_2_O_2_ stress. By using the same method, we identified 245 Atf1 binding sites at the probable promoters in cells prior to H_2_O_2_ treatment. Comparison of enrichment levels between microarray experiments was performed after quantile normalization and signal smoothing of individual ChIP-chip datasets (see Materials and Methods). Clearly, ∼70% (173) of the identified binding sites in cells after H_2_O_2_ treatment were found to overlap (*i.e.*, the distance between the two binding sites was ≤200bps) with those in cells prior to H_2_O_2_ treatment (Figure 1C; Table S1). It was obvious that the enrichment level of the binding sites in cells after H_2_O_2_ treatment was well correlated with that in cells prior to H_2_O_2_ treatment (correlation coefficient = 0.689, *p*-value = <2.2e-16) (Figure 1D).

We found that the median enrichment level of the overlapping Atf1 binding sites in cells under both conditions was clearly higher than that of the non-overlapping binding sites (*i.e.*, the binding sites that were found in cells under only one of two conditions) (3.6–4.0-fold versus 2.4-fold higher than the array median; *p*-value<2.2e-16) (Figure 1D, see *M*). This result indicates that the overlapping binding sites represent the major Atf1 binding sites in the genome. On the other hand, the non-overlapping binding sites were the minor binding sites as it was judged by the low-level enrichment. We therefore concluded that the major Atf1 promoter binding sites were constitutively present before H_2_O_2_ treatment.

A small bZIP factor Pcr1 in *S. pombe* is known to be involved in regulation of transcriptional response to H_2_O_2_, although it may have a minor role [18], [19], [23], [34]. We mapped the Pcr1 binding sites in cells prior to and after H_2_O_2_ treatment using the same method as in the analysis of Atf1 binding sites. The analysis identified that ∼57% (176) of the Pcr1 binding sites in cells after H_2_O_2_ treatment were overlapped with those found in cells prior to H_2_O_2_ treatment (Figure 1E; Table S2). The correlation between enrichment levels of the Pcr1 binding sites that were found in cells prior to and after H_2_O_2_ treatment was apparent (correlation coefficient = 0.803, *p*-value = <2.2e-16) (Figure 1F). Importantly, the median level of the overlapping Pcr1 binding sites was clearly higher than that of the non-overlapping ones (3.9–4.1-fold versus 2.3–2.4-fold higher than the array median; p-value<2.2e-16). Hence, we concluded that, similar to the Atf1, major Pcr1 promoter binding sites were also present before H_2_O_2_ treatment. This conclusion is consistent with, and further supported by, previous analysis of individual loci that are bound by Atf1-Pcr1 heterodimer [18], [24], [34], [43].

### Atf1 and Pcr1 share their promoter binding sites in the genome

**Figure 2:**
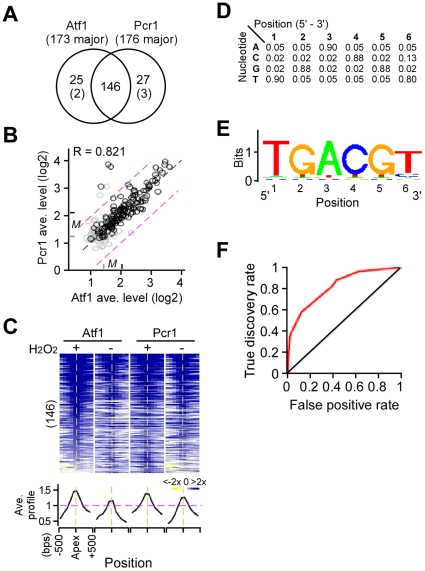
Atf1 and Pcr1 share their major binding sites in the genome. (A) Venn diagram showing the relationship between Atf1 major binding sites and Pcr1 major binding sites. The intersection shows the Atf1/Pcr1 major∶major binding sites. Most of the Atf1 or Pcr1 Major binding sites in the non-intersection part are overlapped with the minor binding sites except for a few (see number in the parentheses) that are not overlapped with either major or minor binding sites indicated. (B) Scatter plot showing the relationship between enrichment levels of Atf1 and Pcr1 at the same loci. The display is identical to Figure 1D. (C) Profile of the Atf1 and Pcr1 occupancies. The heat-maps show the occupancy profile or the level of enrichment within a window of 1 Kb in length (∼33 probes) at each binding site. The profiles of binding sites are aligned at the apex. Average profiles of Atf1 and Pcr1 binding sites in cells prior to and after H_2_O_2_ treatment are shown at the bottom. (D) Position-specific scoring matrix (PSSM) showing the probability of nucleotide at each position. The most enriched motif matrix is shown: the column indicates the nucleotide position of the motif and the row indicates the nucleotide. (E) Motif logo based on the PSSM in (D). (F) ROC curve showing the PSSM scores of the Atf1/Pcr1 binding sequences (the true discovery) versus the randomly selected promoter sequences (the false positives). Black line indicates the randomly occurred motif and the red line indicates the enriched motif in the Atf1/Pcr1 binding sequences.

We wanted to know whether Atf1 and Pcr1 shared their promoter binding sites in the genome. For this reason, the chromosomal locations of the Atf1 and Pcr1 binding sites were compared. All 173 major Atf1 binding sites were found to be overlapped with either major (146) or minor (25) binding sites of Pcr1 except for 2 (Figure 2A; Table S3). Similarly, all 176 major Pcr1 binding sites were overlapped with either major (146) or minor (27) binding sites of Atf1 except for 3. The enrichment level of the Atf1 and Pcr1 binding sites were highly correlated (Figure 2B). It was apparent that the median enrichment level of the major∶major Atf1/Pcr1 common or overlapping binding sites were much higher than that of the major∶minor (or minor∶major) Atf1/Pcr1 binding sites (4.1–4.3-fold versus 2.5–2.7-fold higher than the array median; p-value<2.2e-16). These results indicate that Atf1 and Pcr1 share their major binding sites in the genome, consistent with previous studies on individual genes [22]. A set of the overlapping 146 major∶major Atf1/Pcr1 common binding sites were designated as the major Atf1/Pcr1 (common) promoter binding sites (see Table S3).

Atf1 or Pcr1 enrichment at the major Atf1/Pcr1 binding sites exhibited a typical peak profile (Figure 2C). Its apex location should enrich the Atf1/Pcr1 DNA-binding motif(s). To determine DNA-binding motif(s) that was enriched at the Atf1/Pcr1 binding sites, we performed the motif-discovery scan [44] using the 150bps sequence located at the apexes (see Materials and Methods). The motif-discovery scan showed that the DNA binding sequence represented by the position-specific scoring matrix (PSSM) (Figure 2D) or the logo (Figure 2E) was the most enriched DNA binding motif in the identified Atf1/Pcr1 binding sites. The ROC curve showed that PSSM scores of the Atf1/Pcr1 promoter binding sequences were much higher than that of the randomly selected promoter sequences (Figure 2F), indicating that this motif is preferentially bound by the Atf1/Pcr1 in the whole genome. It was noted that this identified motif was encompassed by the ATF/CREB motif or the *M26* hotspot sequence that are tested for Atf1/Pcr1 binding in the gel-shift assays [12], [17], [45].

Of the 146 Atf1/Pcr1 binding sites, 85 (∼58%) were found in the tandem intergenic regions, locating at the probable promoter of the downstream ORFs. The remaining 61 binding sites (∼42%) were located in the divergent intergenic regions. We found that only 12 out of the 61 divergent intergenic binding sites were shared by the two divergent protein-coding genes. The remaining was found to have either the non-protein-coding genes (e.g., tRNA or 5S rRNA) or the protein-coding gene with the extended upstream sequence (i.e., greater than 1Kb) at one side of the binding sites. Therefore, 146 major Atf1/Pcr1 binding sites were assigned to the probable promoter of the total of 158 genes. It was clear that the Atf1/Pcr1-bound genes enriched for functions such as cellular response to stress or stimulus (GO:51761 and GO:33554; *p*-value<4E-21) and glucose/carbohydrate metabolism (GO:06006 and GO:46164; p-value<4E-11). The top 20 Atf1/Pcr1-bound genes are listed in Table 1 (a complete list of genes in Table S4; see Figure S3 in File S1), most of which are the core environmental stress response (CESR) genes [20].

**Table 1:** List of the top 20 Atf1/Pcr1-bound genes.

No.	Gene name	Atf1 level (log2)^a^	Pcr1 level (log2)^b^	A/P- Rank^c^	CESR^d^	Product^e^
1	SPCC320.03	3.479445	3.957555	1	+	transporter
2	SPACUNK4.19	3.612945	3.77028	2		unknown
3	SPBC1685.13	3.46139	3.850725	3		transporter
4	SPBC660.05	3.37339	3.562445	4	+	unknown
5	SPBC32F12.11|tdh1	3.30439	3.626775	5		glyceraldehyde-3-phosphate dehydrogenase
6	SPAP8A3.04c|hsp9	3.16572	3.74333	6	+	heat shock protein
7	SPACUNK4.15	3.156445	3.4805	7	+	cyclic-nucleotide phosphodiesterase
8	SPAC1039.11c	3.213335	3.35761	8		alpha-glucosidase
9	SPBC1105.13c	3.1415	3.264165	9	+	unknown
10	SPBC1105.14|rsv2	3.1415	3.264165	9	+	transcription factor
11	SPCC63.14	3.198835	3.18039	10	+	unknown
12	SPBC29B5.01|atf1	3.068055	3.25811	11		transcription factor
13	SPCC1672.02c|sap1	3.242055	2.85228	12		switch-activating protein
14	SPCC569.05c	2.856775	3.190445	13		transporter
15	SPAC328.03|tps1	2.92722	3.0935	14	+	trehalose-phosphate synthase
16	SPAC16A10.01	2.95439	3.060225	15	+	unknown
17	SPAC24C9.15c|spn5	2.95439	3.060225	15		septin
18	SPBC354.11c	2.95222	3.047945	16		unknown
19	SPBC215.05|gpd1	2.93778	2.97672	17	+	glycerol-3-phosphate dehydrogenase
20	SPAC17A2.11	2.837775	2.890225	18		unknown

Note: a, Average level of Atf1 enrichment before and after H_2_O_2_ stress; b, Average level of Pcr1 enrichment before and after H_2_O_2_ stress; c, Ranks are based on the average level of Atf1/Pcr1 (A/P) enrichment; d, CESR is based on the study be Chen et al (Chen et al, 2003); e, gene product is mainly based on the S. pombe gene database at www.genedb.org/genedb/pombe. For a complete list of genes whose promoter is bound by Atf1/Pcr1, see Table S4.

### Atf1/Pcr1 bindings are found at their own promoter

**Figure 3:**
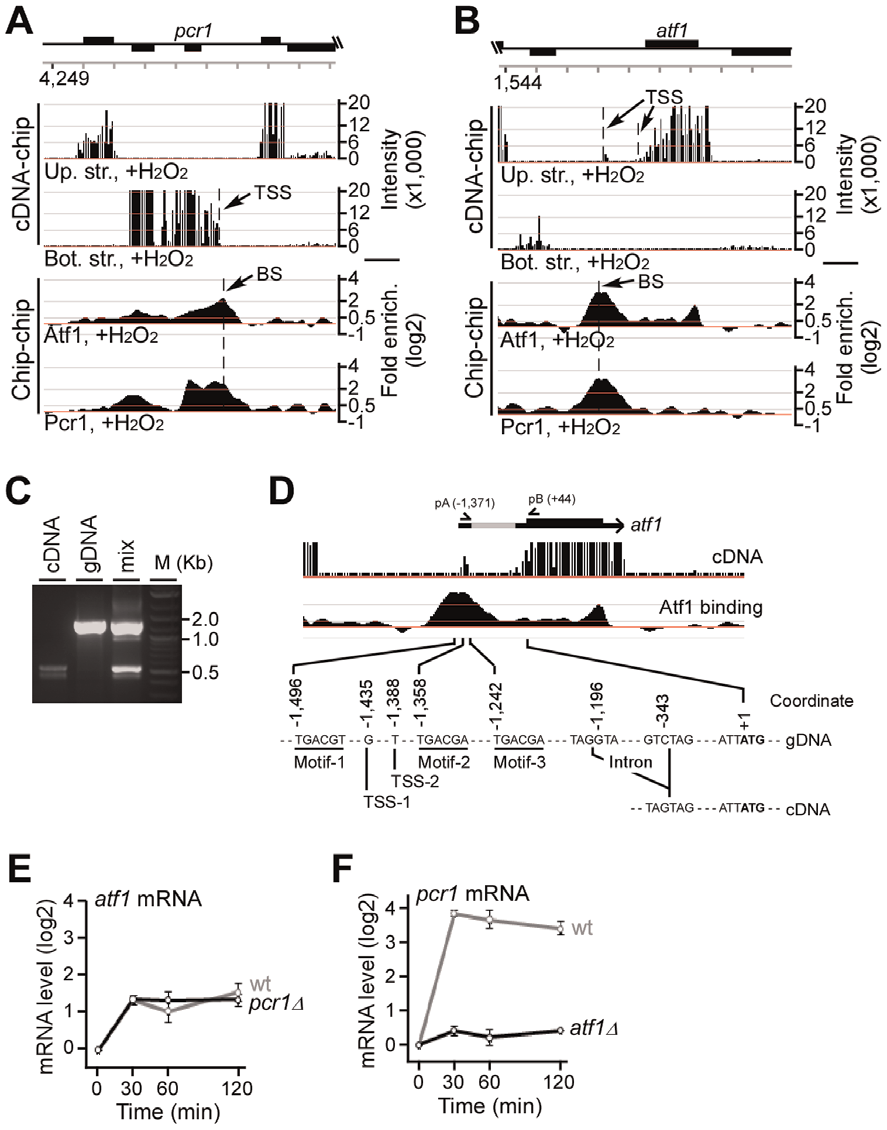
Major Atf1/Pcr1 binding sites are located at their own promoter. (A) Transcription units and Atf1/Pcr1 bindings at the chromosomal region containing *pcr1*. Chromosomal features are indicated at the top. Transcription units in the upper strand (up. str.) or bottom strand (bot. str.) and the Atf1/Pcr1 binding profiles after H_2_O_2_ stress are shown. TSS and BS indicate the transcription start site and binding sites, respectively. (B) Transcription units and Atf1/Pcr1 bindings at the chromosomal region containing *atf1*. Two 5′-end boundaries or TSS are shown. (C) Agarose gel showing the PCR fragments amplified on template cDNA, gDNA (genomic DNA), or the mixture of the two (mix). Molecular weight markers are shown on the left. (D) A magnified view from Figure 3B. *atf1* transcript is shown on the top: the think line indicates the ORF and the grey line indicates the 5′-UTR. The position of primers A and B is indicated. cDNA signals and Atf1 binding profile are shown. At the bottom, 3 motifs at the binding site are indicated as Motif-1 (PSSM score = 18.9) and Motif-2 or 3 (PSSM score = 12.6). TSS predicted by Lantermann et al., (Lantermann et al., 2010) is indicated as TSS-1 and predicted in this study is indicated as TSS-2. The two predicted TSS are very close. The 5′-UTR intron and the start codon (+1) are also indicated. (E) Transcriptional profile of *atf1* in response to H_2_O_2_ treatment. The *atf1* transcript level determined by real-time quantitative PCR is shown at various time points in wild type or *pcr1* 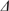 cells. Error bar indicates the variation in three repeated measurements. (F) Transcriptional profile of *pcr1* in response to H_2_O_2_ treatment.

Based on the ORF annotation [41], Atf1/Pcr1 binding sites were found at the probable promoter (upstream intergenic sequences up to 1Kb from the start codon) of *pcr1* gene (Figure 3A), but not *atf1* gene. The promoter binding sites were only recovered for *atf1* gene when TSS annotation [42] was applied (see Materials and Methods), implying that TSS of *atf1* gene was located more than 1Kb upstream of the *atf1* start codon. This result would indicate a positive feedback loop in regulation of transcriptional response by Atf1/Pcr1. To confirm that *atf1* transcript contained the long (>1Kb) 5′-UTR sequence, we examined the transcription start site using the genome tiling microarray (the same tiling microarray used in ChIP-chip mapping of the protein binding sites) that was hybridized by the fluorescent dye-labeled cDNA (see Materials and Methods).

Analysis using segmentation algorithm [46] indicated that the 5′-end of *pcr1^+^* transcript was located at ∼100bps downstream from the Atf1/Pcr1 binding site. On the other hand, the algorithm detected two potential 5′-ends for the *atf1^+^* transcript: one was proximal to the *atf1^+^* coding sequence (∼340bps) and the other was distal to it (∼1.4Kb) (Figure 3B). Interestingly, a major Atf1 or Pcr1 binding site was found to be located at ∼100bps upstream of the distal 5′-end of the *atf1^+^* transcript. This distal 5′-end signal could be resulted from a small transcription unit of ∼100bps in length [47]. Alternatively, the small distal transcript could be part of the *atf1^+^* transcript, provided that there was an intron in the 5′-UTR sequence [42]. To test this possibility, we carried out the PCR assay using sequence-specific primers with either the genomic DNA or cDNA template. It was obvious that a ∼1.4Kb fragment was produced after amplification by PCR on genomic DNA (Figure 3C). On the other hand, a ∼0.5Kb fragment was amplified when cDNA was used as template, indicating that an intron of ∼0.9Kb in size is present in the 5′-UTR sequence of the *atf1^+^* transcript. This was further confirmed by nucleotide sequencing of the PCR amplified products (Figure 3D). Therefore, we confirmed that the promoter of *atf1^+^* contained the major Atf1/Pcr1 binding site. It implies that transcriptional activation of *atf1^+^* and *pcr1^+^* in response to H_2_O_2_ is regulated by the positive feedback loop that could accelerate the transcriptional response.

To examine whether the transcriptional response of *atf1^+^* or *pcr1^+^* required the function of both Atf1 and Pcr1, we performed the real-time quantitative PCR analysis to determine the transcriptional profiles of *atf1^+^* and *pcr1^+^* of various strains in response to H_2_O_2_ treatment. Clearly, both *atf1^+^* and *pcr1^+^* increased their transcript levels in wild type cells after H_2_O_2_ treatment, consistent with the previous report [20], [21]. We found that the response profile of the *atf1^+^* transcription was hardly altered in *pcr1* 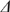 cells, suggesting that Pcr1 is dispensable for the *atf1^+^* transcriptional induction (Figure 3E), although the level of Atf1 protein is reduced in *pcr1* 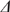 cells [18]. On the other hand, the level of the *pcr1^+^* transcriptional induction was significantly reduced in *atf1* 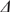 cells, indicating the Atf1 play an essential role in *pcr1^+^* transcriptional response upon H_2_O_2_ treatment (Figure 3F). Pcr1 level is known to be reduced in *atf1* 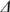 cells [18]. These results suggest that reduced Atf1 level in *pcr1* 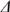 cells is sufficient for self transcriptional activation. In contrast, the reduced Pcr1 level in *atf1* 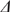 cells is insufficient for self transcriptional activation in response to H_2_O_2_ stress.

### Atf1/Pcr1 is responsible for the high-level transcriptional response upon H_2_O_2_ treatment

**Figure 4:**
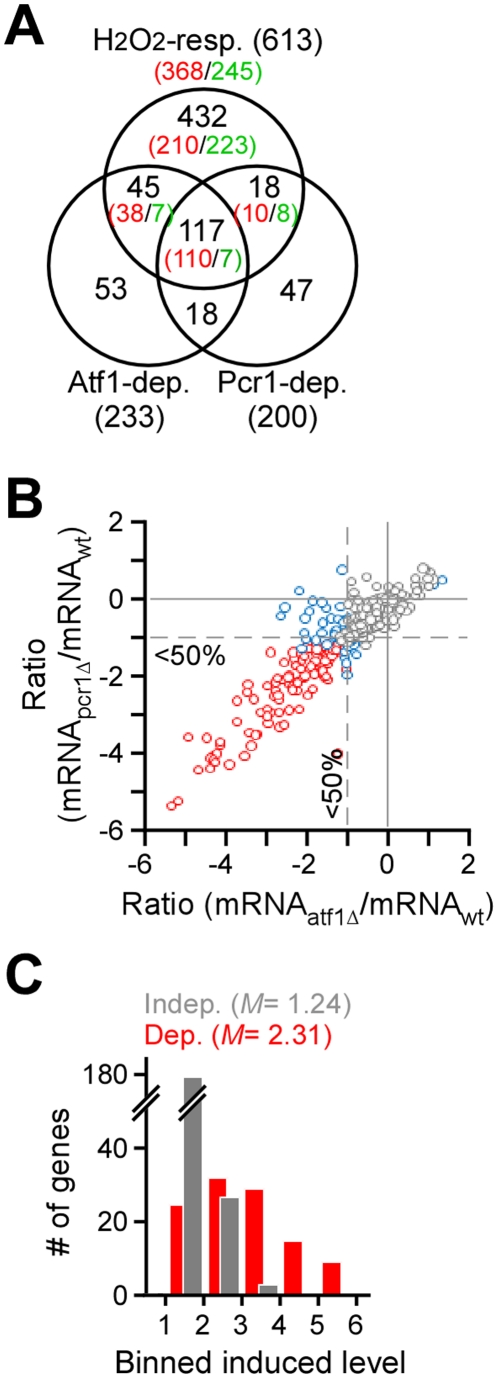
Atf1/Pcr1 is primarily responsible for the high-level transcriptional response to H_2_O_2_. (A) Venn diagram showing the relationship between the Atf1 and/or Pcr1-dependent or independent H_2_O_2_-responsive genes. H_2_O_2_ responsive genes are defined as those whose transcript levels are changed for 2-fold or greater and FDR is less than 3% at least in two consecutive time points examined after H_2_O_2_ stress. The dependent genes are those whose transcript levels are changed for 2-fold or greater and FRD is less than 3% at least in two consecutive time points in mutant cells when compared to the wild type cells (see Materials and Methods). Number in the parentheses indicates the total number of genes. Numbers of induced and repressed genes are indicated in red and green, respectively. (B) Scatter plot showing the ratio between the levels of transcriptional response in *atf1* 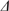 or *pcr1* 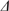 cells and wild type cells. The scale is in log2. Each dot indicates a H_2_O_2_-induced gene. Genes whose transcriptional response is dependent on both Atf1 and Pcr1, one of Atf1 and Pcr1, or none are indicated by red dots, blue dots, or grey dots. Average transcriptional levels of three repeats at 30, 60, and 120min after H_2_O_2_ treatment are used. (C) Histogram showing the number of genes at various levels of average transcriptional induction upon H_2_O_2_ treatment.

We assumed that H_2_O_2_-induced transcriptional profiles of the Atf1 or Pcr1 regulated genes would be disrupted in *atf1* 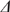 or *pcr1* 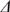 cells. To find the H_2_O_2_-responsive genes that are dependent on the function of Atf1 or Pcr1, we determined transcriptional profiles of *atf1* 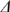, *pcr1* 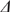, and wild type cells in response to H_2_O_2_ treatment by using the ORF-specific oligonucleotides-based expression microarray in triplicates (see Materials and Methods). SAM analysis of the transcriptional response of wild type cells identified 613 differentially expressed genes: 368 were induced and 245 were repressed (Figure 4A; Table S5). Approximately 73% of the CESR genes [20] were found among the H_2_O_2_-responsive genes (*p*-value<2.2e-16) (see Materials and Methods).

Comparison between transcriptional profiles of wild type and *atf1* 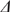 or *pcr1* 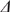 cells revealed that 148 H_2_O_2_-induced genes were significantly disrupted in *atf1* 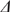 cells and 120 were disrupted in *pcr1* 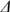 cells, suggesting that the transcriptional response of these genes was dependent on the function of Atf1 or Pcr1. Approximately 92% (110) of the Pcr1-dependent H_2_O_2_-induced genes were also found to be dependent on Atf1 function, indicating that most of the H_2_O_2_-induced Pcr1-depndent genes are co-regulated by both Atf1 and Pcr1 (*i.e.*, the Atf1/Pcr1 heterodimer) (Table 2). Consistent with this, the degree of transcriptional disruption (judged by the ratio between transcriptional response levels in mutant and wild type cells) in *atf1* 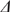 cells was highly correlated with the degree of transcriptional disruption in *pcr1* 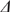 cells (correlation coefficient = 0.88; *p*-value<2.2e-16) (Figure 4B). Interestingly, we found that the genes whose H_2_O_2_-induced transcription profiles were disrupted in atf*1* 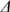 and pcr*1* 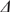 cells appeared to exhibit higher level of transcriptional response when compared to those whose transcriptional induction was not significantly disrupted in mutant cells (*p*-value<2.2E-16) (Figure 4C). This result indicates that Atf1 and Pcr1 are primarily responsible for the regulation of high-level transcriptional induction in response to H_2_O_2_.

**Table 2:** List of the top 25 H_2_O_2_ induced Atf1/Pcr1 dependent genes.

No.	Resp.rank^a^	Gene name	Product
1	1	SPACUNK4.17	NAD binding dehydrogenase family protein
2	2	SPBC16E9.16c	Lsd90 protein
3	3	SPBC3E7.02c|hsp16	heat shock protein
4	4	SPBC365.12c|ish1	LEA domain protein
5	5	SPAC637.03	conserved fungal protein
6	6	SPAC139.05	succinate-semialdehyde dehydrogenase
7	7	SPAC2F3.05c	xylose and arabinose reductase
8	8	SPBC725.03	conserved fungal protein
9	9	SPAC19D5.01|pyp2	tyrosine phosphatase
10	10	SPBC660.05	conserved fungal protein
11	11	SPAC3G6.07	unknown
12	12	SPAC513.02	phosphoglycerate mutase family
13	13	SPAC15E1.02c	unknown
14	14	SPCC338.18	unknown
15	15	SPAC22F8.05	trehalose-phosphate synthase
16	16	SPAC23C11.06c	hydrolase
17	17	SPCC1223.03c|gut2	glycerol-3-phosphate dehydrogenase
18	18	SPBC1773.05c|tms1	hexitol dehydrogenase
19	19	SPBC16D10.08c	heat shock protein
20	20	SPCC1223.02|nmt1	no message in thiamine
21	21	SPBC11C11.06c	unknown
22	22	SPAC22A12.17c	short chain dehydrogenase
23	23	SPCC1739.08c	short chain dehydrogenase
24	24	SPCC191.01	unknown
25	26	SPCC16A11.15c	unknown

Note: a, Responsive (Resp.) rank is based on the average level of transcriptional induction at 30, 60, and 120 min after H_2_O_2_ stress in three repeats when compared to the level at 0 min. For a complete list of the Atf1/Pcr1-dependent H_2_O_2_-induced genes, see Table S10.

Among the top 25 H_2_O_2_-induced genes (based on the average response level of 3 repeats at 30, 60 120min after H_2_O_2_ treatment), all showed disrupted transcription profiles in both *atf1* 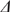 and *pcr1* 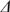 cells except for *srx1^+^*, indicating that their expression response is dependent on the function of both Atf1 and Pcr1, or the Atf1/Pcr1 heterodimer (see Table 2). It was noted that *srx1^+^*, one of the top 25 H_2_O_2_-induced genes whose transcriptional response was Atf1-specific. We found that 38 induced genes were Atf1-specific in response to H_2_O_2_ and only 10 were Pcr1-specific, besides the 110 H_2_O_2_-induced genes that were dependent on both Atf1 and Pcr1 (Table 3). This result implies that Atf1 plays a more prominent role in response to H_2_O_2_ than Pcr1 does, consistent with the observation that *atf1* 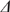 cells display a severe phenotype whereas *pcr1* 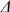 exhibits a weak phenotype under the H_2_O_2_ stress condition (see Figure S1 in File S1) [18], [19], [34] and further supported by the previous analysis of Atf1/Pcr1 binding sites [23].

**Table 3:** List of the top 7 H_2_O_2_ induced Atf1 or Pcr1 specific genes.

No.	Resp.rank^a^	Gene name	Dependency^b^	Product
1	25	SPBC106.02c|srx1	Atf1	sulfiredoxin
2	39	SPBC660.07|ntp1	Atf1	alpha,alpha-trehalase
3	47	SPBC19C7.04c	Atf1	unknown
4	50	SPAC22H12.01c	Atf1	unknown
5	53	SPBC30D10.14	Atf1	dienelactone hydrolase family
6	60	SPCC70.08c	Atf1	methyltransferase (predicted)
7	77	SPBC16A3.02c	Atf1	mitochondrial peptidase (predicted)
8	57	SPAC23G3.03|sib2	Pcr1	ornithine N5 monooxygenase
9	76	SPAC513.07	Pcr1	flavonol reductase/cinnamoyl-CoA reductase family
10	106	SPBC2D10.05|exg3	Pcr1	glucan 1,3-beta-glucosidase
11	178	SPAC343.12|rds1	Pcr1	conserved fungal protein
12	186	SPCC830.08c	Pcr1	ER membrane protein DP1/Yop1
13	199	SPAC4H3.08	Pcr1	3-hydroxyacyl-CoA dehydrogenase
14	216	SPBC3B8.04c	Pcr1	membrane transporter

Note: a, Responsive (Resp.) rank is based on the average level of transcriptional induction at 30, 60, and 120 min after H_2_O_2_ stress in three repeats when compared to the level at 0 min; b, specific dependency is indicated as Atf1 (Atf1-specific) or Pcr1 (Pcr1-specific). For a complete list of the Atf1 or Pcr1-specific H_2_O_2_-induced genes, see Table S11.

### Atf1/Pcr1-bound genes regardless of transcriptional responsiveness to H_2_O_2_ exhibit the high likelihood of requirement for growth fitness in H_2_O_2_

**Figure 5:**
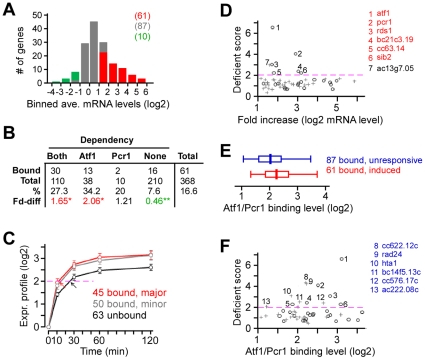
Atf1/Pcr1-bound genes exhibit high likelihood of requirement for resistance and survival to H_2_O_2_. (A) Histogram showing the number of Atf1/Pcr1-bound genes that exhibit various levels of average transcriptional response. Induced, repressed, and unresponsive genes are indicated by red, green, and grey, respectively. (B) Table showing the number of Atf1/Pcr1-bound H_2_O_2_-induced genes in various gene groups indicated. The *p*-value at 0.01 or 0.001 is indicated by 1 asterisk or 2 asterisks. Red and green indicate the enrichment and depletion, respectively. (C) Average expression profile of various gene groups: the Atf1/Pcr1 (major)-bound genes (in red), Atf1/Pcr1 (minor)-bound genes (in grey), and Atf1/Pcr1-unbound genes (in black). The purple dash line indicates the 4-fold increase in level of average transcription. Red and black arrows indicate the time when the average transcription level of the (major) Atf1/Pcr1-bound and unbound genes reaches 4-fold higher than the initial level at 0-min, respectively. (D) Scatter plot showing the level of transcript increase versus the deficient score (the level of requirement for growth fitness). Circle and cross indicate the Atf1/Pcr1-bound H_2_O_2_-induced genes and the unbound induced gene, respectively. (E) Box-plot indicating the distribution of the average level of Atf1 and Pcr1 enrichment at the H_2_O_2_-induced genes (red) and unresponsive genes (blue). (E) Scatter plot showing the level of Atf1/Pcr1 binding versus the deficient score. Circle and cross indicate the Atf1/Pcr1-bound H_2_O_2_-induced genes and Atf1/Pcr1-bound H_2_O_2_-unresponsive genes, respectively.

Genome-wide expression profiling identified that 158 out of 368 H_2_O_2_-induced genes whose response profile was clearly disrupted in *atf1* 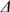 and/or *pcr1* 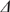 cells, indicating that their transcriptional response is Atf1- and/or Pcr1-dependent (see Figure 4A). Global Atf1/Pcr1-binding profiling also revealed 158 genes whose promoter contained major Atf1/Pcr1 binding sites, suggesting that they are the major genomic targets for Atf1/Pcr1. Given that the bindings were constitutively present before and after H_2_O_2_ stress, some of these Atf1/Pcr1-bound or target genes would not be necessarily involved in response to H_2_O_2_. According to transcriptional response profiles, we found that, among the 158 Atf1/Pcr1-bound genes, 71 (∼46%) were H_2_O_2_-responsive and 85 (∼54%) were not. Of the 71 Atf1/Pcr1-bound H_2_O_2_-responsive genes, 61 were induced, supporting the notion that Atf1/Pcr1 generally play an active role in transcriptional response to H_2_O_2_ (Figure 5A).

We assumed that Atf1/Pcr1-bound H_2_O_2_-induced genes would preferentially show the Atf1/Pcr1-dependent transcriptional profile in response to H_2_O_2_ treatment. To test this assumption, we determined the enrichment level of Atf1/Pcr1-bound H_2_O_2_-induced genes in groups of the Atf1 and/or Pcr1-dependent or independent genes. We found that ∼27.3% of the Atf1/Pcr1-dependent genes was bound by Atf1/Pcr1, which was 1.65-fold of the level by chance (16.6%, *p*-value<0.01) (Figure 5B). And ∼34.2% of the Atf1-specific genes was bound by Atf1/Pcr1, which was more than 2-fold of the level by chance (*p*-value<0.01). In the contrary, we found that 7.6% of the Atf1/Pcr1-independent genes were bound by Atf1/Pcr1, which was less than 50% of the level by chance (*p*-value<0.001). It was noted that no significant enrichment for the Atf1/Pcr1-bound genes in the group of Pcr1-specific genes, because this group contained only 10 genes, 2 of which were bound by Atf1/Pcr1. We therefore concluded that Atf1/Pcr1-bound genes were generally the Atf1/Pcr1-dependent or Atf1-specific H_2_O_2_-induced genes.

Expression profiling indicated that 158 H_2_O_2_-induced genes were dependent on the function of Atf1 and/or Pcr1, of which, 45 were the major Atf1/Pcr1 binding targets and 50 were the minor targets. The remaining 63 genes were not bound by Atf1/Pcr1, implying that these genes are unlikely to be regulated by Atf1/Pcr1 directly. We assumed that the decay rate for all transcripts was similar and proposed that the induction of these Atf1/Pcr1-unbound genes were the indirect result of the Atf1/Pcr1-regulated transcriptional response to H_2_O_2_ treatment. This is because, during response to H_2_O_2_ stress, the induction of some of the Atf1/Pcr1 direct targets (or the bound induced genes) such as *cgs2* [33] could be responsible for the induction of the Atf1/Pcr1-unbound genes. If this was the case, we would see a time delay in transcriptional response for these unbound induced genes (i.e., the indirectly regulated genes) when compared to that of the bound induced genes (i.e., the directly regulated genes). For this reason, we examined the time that was needed to reach the level of 4-fold increase in the average transcript level of the Atf1/Pcr1-bound induced and Atf1/Pcr1-unbound induced genes. It took a little bit more than 10min for the Atf1/Pcr1-bound H_2_O_2_-induced genes to reach the level of 4-fold increase (Figure 5C). On the other hand, it took nearly 30min to reach that level for the Atf1/Pcr1-unbound H_2_O_2_-induced genes. This result support the idea that some of the Atf1/Pcr1-bound Atf1/Pcr1-dependent genes are responsible for the transcriptional response the Atf1/Pcr1-unbound Atf1/Pcr1-dependent genes. Furthermore, we tested if the Atf1/Pcr1 indirectly regulated (i.e., Atf1/Pcr1-unbound) H_2_O_2_-induced genes would exhibit a lower probability of requirement for growth fitness under the H_2_O_2_ stress conditions than did the Atf1/Pcr1 directly regulated (i.e., Atf1/Pcr1-bound) ones. To this end, we selected the 60 mutant strains bearing a deletion allele of the Atf1/Pcr1-dependent H_2_O_2_-induced genes available from the Bioneer haploid deletion strain set (see Materials and Methods). Among these strains, 26 contained a deletion allele of the Atf1/Pcr1-bound genes and 34 contained the Atf1/Pcr1-unbound genes deletion allele.

Mini-culture growth curve analysis was performed to determine if the deletion strains exhibited growth defect in presence of H_2_O_2_. We found that half-maximal time *T_50_* (i.e., the time required for culture to reach half-maximal concentration) was inversely proportional to the initial culture concentration (see Figure S4 in File S1). Thus, the difference (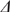 *T_50_*) between *T_50_* of cultures in media supplemented with and without H_2_O_2_ would indicate the level of growth defect under H_2_O_2_-induced stress condition. We defined the normalized 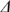 *T_50_* of individual deletion strains (i.e., 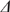 *T_50_* of mutant strains was divided by 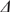 *T_50_* of the wild type) as deficient score. The strain was designated as H_2_O_2_-sensitive if its deficient score was 2 or greater. The mini-culture growth curve assay identified 6 out of 26 (∼23%) Atf1/Pcr1-bound-gene deletion strains that exhibited H_2_O_2_ sensitivity (Figure 5D; Table S6). On the other hand, only 1 out of 34 (∼2.9%) Atf1/Pcr1-unbound-gene deletion strains showed H_2_O_2_ sensitivity. Clearly, this result supports the idea that Atf1/Pcr1 directly regulated H_2_O_2_-induced genes play a major role but not the Atf1/Pcr1 indirectly regulated H_2_O_2_-induced genes in regulation of growth fitness in H_2_O_2_ stress conditions (*p*-value = 0.013).

We found that 87 out of 158 Atf1/Pcr1-bound genes were not responsive to H_2_O_2_ treatment (see Figure 5A, grey bars). The Atf1/Pcr1 binding level at these genes were slightly lower than those at the induced genes (Figure 5E), suggesting that the primary role for Atf1/Pcr1 is to induced transcriptional response to H_2_O_2_. Since the Atf1/Pcr1 bindings were present before H_2_O_2_ treatment, many of the Atf1/Pcr1-bound genes might be involved in response to other stress factors but not H_2_O_2_. Therefore, we wanted to know if the Atf1/Pcr1-bound H_2_O_2_-unresponsive genes (13 bound unresponsive genes were excluded because their basal transcription was significantly altered in *atf1* 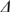 and/or *pcr1* 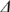 cells) would show a low likelihood of requirement for H_2_O_2_ resistance and survival as the Atf1/Pcr1-unbound H_2_O_2_-induced genes. Surprisingly, mini-culture growth curve assay indicated that ∼25% (6 out of 24 strains tested) of the random selected mutant strains bearing a deletion allele of the Atf1/Pcr1-bound H_2_O_2_-unresposnive genes were sensitivity to H_2_O_2_ (Figure 5F), displaying a high likelihood of requirement as the Atf1/Pcr1-bound H_2_O_2_-induced genes. This result indicates that Atf1/Pcr1-bound genes, regardless of transcriptional induction by H_2_O_2_, exhibit the high likelihood of requirement for growth fitness in H_2_O_2_ stress condition.

### Sty1 is primarily recruited at the Atf1/Pcr1 binding sites upon H_2_O_2_ treatment

To determine the genomic binding profiles of Sty1, we performed the ChIP-chip analysis of Sty1 in cells prior to and after H_2_O_2_ treatment. The analysis showed that there are ∼230 promoter binding sites in cells after H_2_O_2_ treatment. On the other hand, less than 100 promoter binding sites were found in cells prior to H_2_O_2_ treatment. No apparent correlation between levels of Sty1 binding in cells prior to and after H_2_O_2_ treatment was found (see Figure S5 in File S1). This result suggests that the Sty1 recruitment are primarily induced by H_2_O_2_ stress, consistent with the observation that Sty1 was translocated into nucleus upon H_2_O_2_ treatment [15], [19], [27]. Although about half of the identified Sty1 binding sites in cells prior to H_2_O_2_ stress were found to be overlapped in the two independent repeats, hardly any correlation was found between the enrichment levels of Sty1 binding sites in the two repeats (see Figure S6 in File S1), suggesting that the Sty1 binding in the genome prior to H_2_O_2_ stress is less specific. Hence, we determined the Sty1 binding sites in cells after H_2_O_2_ based on two independently repeated ChIP-chip experiments.

**Figure 6:**
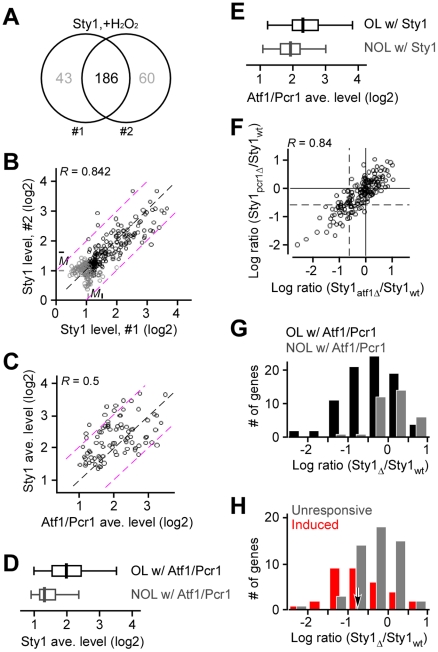
Atf1/Pcr1 binding sites are the major targets of the Sty1 recruitment upon H_2_O_2_ treatment. (A) Venn diagram showing the relationship between the Sty1 binding sites found in two independently repeated ChIP-chip experiments. (B) Scatter plot showing the relationship between the levels of Sty1 recruitment in two repeats. Black and grey dots indicate the overlapping and non-overlapping binding sites in two repeats. The diagonal and two-fold lines are indicated in black and purple, respectively. (C) Scatter plot showing the relationship between the levels of average Atf1/Pcr1 enrichment and average Sty1 enrichment at the 89 loci containing both major Atf1/Pcr1 and major Sty1 binding sites. The diagonal and two-fold lines are indicated in black and purple, respectively. (D) Box-plot showing the average levels of Sty1 enrichment at the loci that are overlapped with (OL w/) major Atf1/Pcr1 (in black) or not overlapped with (NOL w/) Atf1/Pcr1 (in grey) binding sites. (E) Box-plot showing the average levels of Atf1/Pcr1 enrichment at the loci that are overlapped with (OL w/) major Sty1 (in black) or not overlapped with (NOL w/) Sty1 (in grey) binding sites. (F) Scatter plot showing the ratio between Sty1 enrichment levels in *atf1* 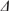 or *pcr1* 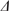 and wild type cells. The solid line indicates the median and the dash line indicates the ratio at 50% reduction (−0.585 in log2 scale). (G) Histogram showing the number of Sty1-bound genes that exhibit various ratios between Sty1 enrichment levels in *atf1* 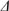/*pcr1* 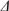 and wild type cells. Sty1-bound genes that are also Atf1/Pcr1-bound (OL w/) or not bound (NOL w/) are indicated in black or grey. (H) Histogram showing the number of Sty1-bound Atf1/Pcr1-bound genes that exhibit various ratios between Sty1 enrichment levels in *atf1* 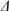/*pcr1* 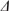 and wild type cells. Genes that are induced or unresponsive upon H_2_O_2_ treatment are indicated by red or grey bars. The arrow indicates the cutoff for the Atf1/Pcr1-dependently Sty1-recrutied genes.

Approximately 186 (∼81%) Sty1 binding sites were identified in cells after H_2_O_2_ treatment in two independently repeated ChIP-chip experiments (Figure 6A). The enrichment level of Sty1 binding sites was well correlated between the two experiments (correlation coefficient = 0.84; *p*-value<2.2e-16) (Figure 6B; Table S7). The Sty1 enrichment level at the overlapping sites was clearly higher than that of the non-overlapping ones (*p*-value<2.2e-16). We thus designated the overlapping sites in the two repeats after H_2_O_2_ stress as the major Sty1 recruitment sites. Of the 186 major Sty1 recruitment sites, 152 (∼82%) were found to overlap with the Atf1/Pcr1 binding sites (89 overlapped with the major Atf1/Pcr1 common binding sites and 63 with Atf1 and/or Pcr1 binding sites), indicating that Sty1 is primarily recruited at the Atf1/Pcr1 binding sites in the genome induced by H_2_O_2_ treatment.

Among the 89 major Sty1 binding sites that overlapped with the major Atf1/Pcr1 binding sites, a significantly correlation was found between the enrichment levels of Sty1 and Atf1/Pcr1 (i.e., the average level of Atf1 and Pcr1) (correlation coefficient = 0.5, *p*-value = 4.61e-07) (Figure 6C). This result suggests that the level of Sty1 recruitment is correlated with the enrichment level of the Atf1/Pcr1 binding. Consistent with this, we found that the median enrichment level of Sty1 at the binding sites overlapped with the major Atf1/Pcr1 binding sites was higher than that of the Sty1 at the binding sites non-overlapped with the Atf1/Pcr1 binding sites (4-fold versus 2.3-fold higher than the array median; *p*-value = 3.03e-08) (Figure 6D). Likewise, of the 146 major Atf1/Pcr1 binding sites, less than 30% were found no Sty1 recruitment. The median Atf1/Pcr1 enrichment level at the Sty1-recruitment sites was higher than that of the non-Sty1-recruitment sites (4.7-fold versus 3.6-fold higher than the array median; *p*-value = 8.96e-04) (Figure 6E). This result indicates that Atf1/Pcr1 binding in the genome is the primary target for Sty1 recruitment upon H_2_O_2_ treatment.

To test if the recruitment of Sty1 was dependent on the function of Atf1/Pcr1, we performed ChIP-chip analysis of Sty1 in *atf1* 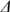 or *pcr1* 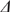 cells after treatment with H_2_O_2_. Atf1/Pcr1 dependency of Sty1 recruitment was estimated by the ratio between the enrichment levels of Sty1 in *atf1* 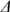 or *pcr1* 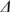 and wild type cells. Clearly, the ChIP-chip analysis showed that the ratio between Sty1 levels in *atf1* 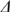 and wild type cells was significantly correlated with that between Sty1 levels in *pcr1* 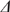 and wild type cells (correlation coefficient = 0.84, *p*-value<2.2e-16) (Figure 6F), indicating that the Sty1 recruitment requires both Atf1 and Pcr1. This is consistent with the notion that Atf1 and Pcr1 form heterodimer in vitro or in vivo [22], [24], [33]. We thus utilized the ratio between the Sty1 levels in mutants (average of *atf1* 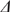 and *pcr1* 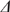) and wild type cells (average of two independent repeats) to estimate the dependency of Sty1 recruitment on Atf1/Pcr1. Not surprisingly, the level of Sty1 recruitment at the Atf1/Pcr1-unbound loci showed no apparent reduction in *atf1* 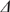 or *pcr1* 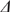 cells (Figure 6G, see grey bars). On the other hand, the Sty1 recruitment at many Atf1/Pcr1-bound loci showed the ∼25% reduction when compared to the array median (Figure 6G, see black bars). Though the level of reduction was not dramatic, it was considered to be statistically significant when compared to those at the Atf1/Pcr1-unbound loci (*p*-value = 4.77e-06).

Many Atf1/Pcr1-bound genes were found to be unresponsive to H_2_O_2_ treatment (see Figure 5A). We therefore wanted to know if the Atf1/Pcr1-dependency of Sty1 recruitment would preferentially occur at the H_2_O_2_-induced genes. To this end, 89 Sty1-recruited Atf1/Pcr1-binding sites were assigned to 101 genes (see Materials and Methods) (Table S8). Among the 101 Sty1-recrutited genes, 44 were H_2_O_2_-responsive (39 were induced and 5 were repressed) genes and the remaining 57 were H_2_O_2_-unresponsive genes. Notably, the ratio between Sty1 recruitment levels in *atf1* 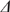 or *pcr1* 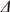 and wild type cells at the H_2_O_2_-induced genes was lower than that at the unresponsive genes (0.56 versus 0.92; *p*-value<0.001) (Figure 6H). This result indicates that the dependence of Sty1 recruitment on Atf1 or Pcr1 is apparent at the H_2_O_2_-induced genes, but not at the H_2_O_2_-uresponsive genes.

We defined that the Sty1 recruitment was Atf1/Pcr1-dependent if the ratio between the Sty1 recruitment levels in *atf1* 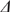 or *pcr1* 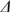 and wild type cells was less than 0.6. Hence, 31 out of 101 Sty1-recruited genes exhibited Atf1/Pcr1-dependent recruitment (one of the 101 genes was not presented in the expression microarray) (Table 4). Clearly, 24 out of 31 Atf1/Pcr1-dependent Sty1-recruited genes were found in the group of 39 Atf1/Pcr1-bound Sty1-recruited H_2_O_2_-induced genes, which was 2-fold higher than the background level (∼30.7%) (*p*-value = 1.81e-05). On the other hand, only 7 Atf1/Pcr1-dependent Sty1-recruited genes were found in the group of 57 Atf1/Pcr1-bound Sty1-recruited H_2_O_2_-unresponsive genes, which was only 40% of the background level (*p*-value = 1.8e-04). We hence conclude that Atf1/Pcr1-dependent Sty1 recruitment primarily occurred at the Atf1/Pcr1-bound H_2_O_2_-induced genes.

**Table 4:** List of the 31 genes exhibit the apparent Atf1/Pcr1-dependent recruitment for Sty1.

No.	Sty1 rank^a^	Gene name	Resp. rank^b^	Sty1 level^c^	Ratio (mut/wt)^d^	Resp. depend.^e^	Atf1/Pcr1 rank^f^
1	11	SPBC1683.01	217	2.94	0.54	Atf1	47
2	18	SPBC1105.13c	52	2.71	0.57	Atf1/Pcr1	9
3	18	SPBC1105.14|rsv2	114	2.71	0.57	Atf1	9
4	21	SPAC1751.01c|gti1	44	2.61	0.41		90
5	23	SPAC343.12|rds1	178	2.53	0.44	Pcr1	33
6	25	SPAP8A3.04c|hsp9	102	2.49	0.35	Atf1/Pcr1	6
7	32	SPAC25B8.12c	NA	2.35	0.59	Pcr1	30
8	40	SPBC660.05	10	2.26	0.31	Atf1/Pcr1	4
9	43	SPCC794.12c|mae2	NA	2.21	0.56		31
10	44	SPAC22F8.05	15	2.20	0.25	Atf1/Pcr1	50
11	48	SPAC328.03|tps1	87	2.16	0.39		14
12	49	SPAC16A10.01	98	2.15	0.38	Atf1	15
13	49	SPAC24C9.15c|spn5	NA	2.15	0.38		15
14	53	SPACUNK4.17	1	2.09	0.46	Atf1/Pcr1	26
15	59	SPACUNK4.15	68	2.03	0.47	Atf1/Pcr1	7
16	61	SPBC21C3.19	62	2.01	0.43	Atf1/Pcr1	70
17	63	SPBC29B5.01|atf1	175	1.99	0.55	Atf1	11
18	65	SPBPB21E7.08	NA	1.96	0.20		69
19	69	SPAC23H3.15c	30	1.86	0.30	Atf1/Pcr1	36
20	69	SPAC25H1.02|jmj1	155	1.86	0.30	Atf1/Pcr1	36
21	70	SPCC757.07c|ctt1	137	1.85	0.59	Atf1	75
22	79	SPAC19D5.01|pyp2	9	1.73	0.58	Atf1/Pcr1	27
23	83	SPAC3A11.07	NA	1.67	0.52		107
24	85	SPCC1322.07c	64	1.66	0.37	Atf1/Pcr1	32
25	85	SPCC1322.08|srk1	54	1.66	0.37	Atf1/Pcr1	32
26	89	SPAC13F5.03c	38	1.61	0.58	Atf1/Pcr1	41
27	106	SPBP4G3.02|pho1	NA	1.52	0.46		137
28	135	SPBC713.11c|pmp3	NA	1.28	0.50	Atf1/Pcr1	96
29	141	SPCP31B10.06	61	1.25	0.43	Atf1/Pcr1	24
30	159	SPAC32A11.02c	56	1.18	0.56	Atf1/Pcr1	54
31	162	SPAC8C9.03|cgs1	71	1.16	0.46	Atf1/Pcr1	44

Note: a, Sty1 rank is based on the level of Sty1 recruitment; b, responsive rank is based on the level of transcription induction (unresponsive genes are indicated as not applicable or NA); c, Sty1 level is in log2 scale; d, ratio between Sty1 level in mutants and level in wild type cells; e, Response dependency (depend.) indicates the dependence of transcriptional responses; f, Atf1/Pcr1 rank is based on the average level of Atf1 and Pcr1 enrichment.

## Discussion

The evolutionarily conserved MAPK Sty1 and bZIP transcription factor Atf1 in fission yeast play a major role in response to various environmental stress factors [9], [26]. In this study, we demonstrate that the major Atf1 binding sites in the genome are present before and after H_2_O_2_ stress (Figure 1C–D). The Atf1 binding partner, a small bZIP protein Pcr1, is found to share most of the Atf1 binding sites (Figure 2A–B), though the phenotype of *pcr1* 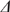 is much weaker than that of *atf1* 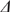 upon H_2_O_2_ stress (see Figure S1 in File S1) that has been reported [18], [23], [34]. Because Atf1 or Pcr1 alone can also bind DNA [22], it is therefore conceivable that Atf1 and Pcr1 are able to act as either heterodimer or homodimer to regulate transcription during response to H_2_O_2_.

Phenotypic assessment indicates that the bound-induced genes have a higher likelihood of requirement for growth fitness under the H_2_O_2_-induced stress condition than do the unbound-induced genes (Figure 5D), indicating that the bound-induced genes are the primary set of genes that are regulated by Atf1/Pcr1 in response to H_2_O_2_. Indeed, the unbound induced genes show a delayed response when compared to the bound induced genes (Figure 5C), suggesting that the unbound induced genes may be controlled by the product of the bound induced genes such as *cgs2* [33], but not regulated directly by Atf1/Pcr1. Interestingly, of the Atf1/Pcr1-dependent H_2_O_2_-induced genes, Atf1/Pcr1-bound ones show a severer reduction in transcriptional response in *atf1* 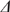 cells than that in *pcr1* 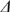 (see Figure S7 in File S1). Furthermore, we found that 13 Atf1-specific H_2_O_2_-induced genes are bound by Atf1/Pcr1 but only 2 Pcr1-specific genes are bound by Atf1/Pcr1 (Figure 5B). These results may explain why *atf1* 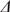 exhibits a severer phenotype than the *pcr1* 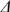.

It has been shown that *nmt1* promoter-controlled Atf1 in *pcr1* 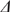 background or Pcr1 in *atf1* 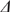 background can localize to nucleus, indicating that their nucleus localization does not require the Atf1/Pcr1 heterodimer function [34]. It is therefore most likely that Atf1 or Pcr1 can bind to their genomic targets without formation of heterodimer [22]. If this is true, one would expect that Atf1 or Atf1 homodimer is more active than Pcr1 homodimer in transcriptional response to H_2_O_2_ stress, while the heterodimer is the most active form. Alternatively, Atf1 could form alternative heterodimer more efficiently than does Pcr1. It has been shown that Pcr1 is only required for localization of the Atf1/Pcr1 heterodimer to the recombination hotspot *ade6* [48]. Tethering of Atf1 through a heterologous DNA binding domain to *ade6* without Pcr1 can promote meiotic recombination [48], [49]. Therefore, we conclude that Atf1 plays a more important role in transcriptional response to H_2_O_2_ stress than does Pcr1.

In this study, we show that Atf1/Pcr1 bind at the promoter of both *atf1^+^* and *pcr1^+^* genes (Figure 3), indicating a positive feedback loop in transcriptional regulation of *atf1^+^* and *pcr1^+^* that has been hypothesized by others [14]. Clearly, transcriptional response of *pcr1^+^* is largely diminished in *atf1* 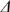 cells, consistent with the previous report [18]. In contrast, the transcriptional response of *atf1^+^* is hardly affected in *pcr1* 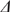 cells, although the level of Atf1 protein is impaired in *pcr1* 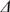 cells [18]. This result suggests that the reduced level or Atf1 protein in *pcr1* 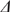 cells does not affect the *atf1* self transcriptional response.

Hundreds of genes are found to be differentially transcribed upon H_2_O_2_ stress in this study (see Figure 4A). Approximately 40% (158) of the induced genes and 10% (24) of the repressed genes are dependent on the function of Atf1 and/or Pcr1. On the other hand, ChIP-chip analysis shows that 45 out of 158 induced dependent genes and 2 out of 24 repressed dependent genes are bound by Atf1/Pcr1 at the promoter. These results indicate that Atf1/Pcr1 is primarily involved in regulation of many induced genes but not repressed genes upon H_2_O_2_ stress. It has been shown that the Atf1 binding levels increased at the promoter of the stress-induced *gpd1* and *hsp9* genes after osmotic stress [18]. Although Atf1 binding levels are not apparently increased at the promoter of *gpd1* and *hsp1* after H_2_O_2_ stress in this study, we do observed that the levels of Atf1 binding at the induced genes but not at the unresponsive genes appear to be increased after stress (*p*-value = 0.013) (see Figure S8 in File S1). However, the tendency for binding level increase at the induced genes is not obvious for Pcr1. This probably is a result of stabilization of Atf1 by Pcr1 upon H_2_O_2_ stress, as proposed by Lawrence et al. [18], at the induced genes but not at the unresponsive genes.

We show here that majority of the Atf1/Pcr1-bound genes are not responsive to H_2_O_2_ stress (Figure 5A). Significantly, these unresponsive genes display a similar likelihood of requirement for growth fitness under H_2_O_2_-induced stress condition as the bound-induced genes (Figure 5F). We propose that the Atf1/Pcr1-bound H_2_O_2_-unresponsive genes are involved in response to other stress factors. Thus, exposure of cells to other stress factors that induce the Atf1/Pcr1-bound H_2_O_2_-unresponsive genes would enable the cells to adapt to the H_2_O_2_ stress conditions. Exposure of cells to one stress may enable them to adapt to other stress conditions. It is therefore conceivable that the Sty1-Atf1 pathway could play a role in the acquired stress adaptation in fission yeast.

In this study, we show that, unlike Atf1/Pcr1, Sty1 is recruited to the promoter after H_2_O_2_ treatment (see Figure S5 in File S1). Our analysis indicates that Sty1 is primarily recruited to the Atf1/Pcr1 binding sites in the genome (Figure 6D), indicating that Atf1/Pcr1 is a major nuclear target for Sty1. Sty1 recruitment shows the apparent dependence on Atf1/Pcr1 at the Atf1/Pcr1-bound H_2_O_2_-induced genes but not at the bound-unresponsive genes (Figure 6H). This result implies that other factors are bound at the promoter of the Atf1/Pcr1-bound H_2_O_2_-unresponsive genes. We propose that these unknown factors would play at least two roles: suppression of Atf1/Pcr1 activity upon H_2_O_2_ treatment and recruitment of Sty1 when Atf1 or Pcr1 is absent. Further studies on transcription factors involved in response to other stresses will allow us to identify these factors. In conclusion, our analysis of genomic binding profiling of MAPK Sty1 and bZIP transcription factors Atf1/Pcr1, global transcription profiling of cells in response to H_2_O_2_ tress, and phenotypic assessment of ∼90 deletion strains provide insight into mechanisms for global regulation of transcriptional response to H_2_O_2_ by the Sty1-Atf1/Pcr1 pathway in fission yeast.

## Materials and Methods

### DNA, strains and culture manipulations

The strains used in this study are shown in Table 5. Deletion or epitope-tagging strains were constructed as previously described [50]. Log-phase growth cells (OD600 = ∼0.3–.0.4) were subject to the treatment with 0.5mM H_2_O_2_ (Sigma-Aldrich, St. Louis, MO) for various period of time for ChIP-chip binding analysis (0 and 30min) or transcription profiling analysis (0, 10, 30, 60, and 120min). Cells from ∼20ml cultures at various time points was spun out, snap-chilled in liquid nitrogen, and stored at −80C for later RNA extraction using hot-phenol protocol [51]. For ChIP experiment, cells from ∼200ml cultures were treated with 1% formaldehyde (v/v) (Sigma-Aldrich) for 10min before harvest. Sequence-specific primers (primer A: 5′-GTTGGATCTGAATTACGAATTCTC-3′ and primer B: 5′- AGTACTGGAAGCAGTAGCATTACC-3′) were used in PCR amplification using either genomic DNA or cDNA.

**Table 5:** List of strains used for microarray analyses in this study.

ID	Relevant genotype	Comment
LJY188	*h^-^ leu1-32 ura4-D18::ura4^+^*	Lab stock
LJY2257	*h^-^ leu1-32 ura4-D18 atf1* 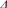 *::ura4^+^*	This Study
LJY2261	*h^-^ leu1-32 ura4-D18 pcr1* 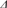 *::ura4^+^*	This Study
LJY2178	*h^-^ leu1-32 ura4-D18 atf1^+^-3HA-6His::ura4^+^*	This Study
LJY2223	*h^-^ leu1-32 ura4-D18 pcr1^+^-3HA-6His::ura4^+^*	This Study
LJY1894	*h^-^ leu1-32 ura4-D18 sty1^+^-3HA-6His::ura4^+^*	This Study
LJY2851	*h^-^ leu1-32 ura4-D18 sty1^+^-3HA-6His::LEU2 atf1* 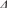 *::ura4^+^*	This Study
LJY2852	*h^-^ leu1-32 ura4-D18 sty1^+^-3HA-6His::LEU2 pcr1* 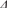 *::ura4^+^*	This Study

### Phenotypic assessment

Plating assay was applied for testing sensitivity to H_2_O_2_ in various HA-tagged strains used in ChIP-chip analysis. The 10-fold serial diluted cells were spotted on plates supplemented with or without H_2_O_2_ and incubated for 2–4 days at 30C. The Bioscreen-C system (Growth Curves USA Inc., Piscataway, NJ) was used to determine the mini-culture growth curve for quantitative measurement of H_2_O_2_ sensitivity in various gene deletion strains (Bioneer Corp., Daejeon, South Korea). The timing at the half-maximal-concentration (*C_half-max_*) was found to be proportional to the concentration of the initial cultures (see Figure S4A in File S1). Cells under the stress condition would take longer time (*T_50-stress_*) to reach the *C_half-max_* when compared to the *T_50-optimal_* of cells under the optimal condition. The time difference (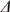 *T_50_* = *T_50-stress_*–*T_50-optimal_*) in reaching the *C_half-max_* between stress and optimal conditions was proportional to the level of sensitivity to H_2_O_2_ in *atf1* 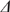 and *pcr1* 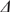 cells (see Figure S4B in File S1). The ratio (average of at least 3 repeats) between the 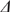 *T_50_* in the deletion and wild type strains was defined as the deficient score. Genes whose disruption confers the sensitivity to H_2_O_2_ would be functionally required for growth under the H_2_O_2_ stress condition. The deficient score was proportional to the level of the requirement for growth under stress.

### Chromatin immunoprecipitation (ChIP)

Cell lysate was prepared using the Fast Prep glass-beads beater (Bio 101, Carlsbad, CA) for 5 30m-bursts with a interval of minimal 2min. The lysate was further homogenized 3 times at 30% of maximal strength for 30-sec sonication (Branson Power Company, Danbury, CT). Part of the resulting lysate was used for DNA extract (e.g., WCE-DNA) and the remaining was mixed with anti-HA antibody-coupled agarose beads (Santa Cruz Biotechnology Inc., Santa Cruz, CA) to enriched DNA molecules that are bound by the HA-tagged proteins (ChIP-DNA). Both ChIP-DNA and WCE-DNA were linear amplified by the GenomePlex amplification kit (Sigma-Aldrich) before labeling for microarray hybridization (second chip).

### Labeling of nuclear acids and hybridization of microarrays

Linear-amplified DNA was labeled with amino-allyl-dUTP by random priming using the BioPrime DNA Labeling kit, (Invitrogen Co.). Amino-allyl-dUTP-containing DNA was coupled to the Cyanine dyes Cy5 or Cy3 (Amersham). Cy5-labeled ChIP-DNA and Cy3-labeled WCE-DNA were co-hybridized to the genome-tiling DNA microarrays. In transcriptional profile analysis, cDNA was synthesized from the hot-phenol extracted RNA using the Superscript II (Invitrogen Co., Carlsbad, CA) and poly(dT) primers in presence of amino-allyl-dUTP. Cy5-labeled sample cDNA and Cy3-labeled common reference cDNA (e.g., log-phase growth wild type cells) were co-hybridized to the ORF-specific DNA microarrays. The washing and scanning of the microarray were done as previously described [51].

### Microarray design and data acquisitions

High-resolution genome tiling microarrays were designed to cover both strand of the *S. pombe* genome with ∼380 thousands 50-mer oligonucleotides tiled 17bps at alternate strand. The customized microarrays were manufactured using the mask-less NimbleGen technology (Roche NimbleGen Inc., Madison, WI). Approximately 10 thousand 50-mer oligonucleotides representing ∼5,000 ORFs were spotted by the Arrayer (GeneMachines, San Carlos, CA) on the poly-Lysine coated glass slides and post-processed as described previously [51].

Hybridized microarray slides were scanned using the Axon GenePix 4000B scanner (Molecular Devices, Sunnyvale, CA) at a resolution of ∼5 µm at 532 and 635 nm. The NimbleScan 3 (Roche NimbleGen Inc.) software and GenePix Pro7 (Molecular Devices) were used to acquire the fluorescence signals of array features in NimbleGen microarrays and Spotted ORF-specific microarrays, respectively. LOWESS normalization [52], [53] was subsequently applied to all microarray data.

### Identification of the significantly enriched region (SER)

Enrichment level at various microarray features/probes is indicated by the ratio between Cy5 (enriched) and Cy3 (non-enriched) signals in log2 scale. All ChIP-chip microarray data were quantile normalized. Individual occupancies (or SER) were assessed in a moving window of 9-probes (or ∼300bps) in length. Binomial test was applied to determine the probability of probe that passes the threshold within the window: 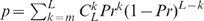, where *Pr = N_R≥MAD_R_/N*, *N* = 358,000, the total number of probes representing the genome sequences in the array; *MAD_R = Median(Ri)+n×MAD(Ri)*, *n* = 2.5, *R* is the enrichment level of the probe, *Ri* is the level of the *i*th probe along the chromosome, *m* is the number of probes whose level was greater than the *MAD_R* in a window of *L* numbers of probes in size, where *L* = 9. Occupancies were defined as the regions where there were at least 4 consecutive probes whose enrichment was greater than *MAD_R* at *p*-value of 0.001 or less. Enrichment level and binding site (i.e., position of the Apex) of individual occupancies were determined after 3 round linear smoothing of microarray data at a window of 5 probes.

Binding sites were assigned to the probable promoter (it was defined from up to 1Kb upstream intergenic sequences to 200 bps downstream coding sequences from the start codon [41] or from up to 150bps upstream intergenic sequences to 150bps downstream from the TSS [42] of individual protein coding genes. A small fraction of occupancies are found in the intragenic sequences. In this study, intragenic occupancies are not discussed. However, genes whose intragenic sequences contain Atf1 and/or Pcr1 binding sites are listed in Table S9. Two binding sites are overlapping if the distance between the two is less than 200bps. Major binding sites are overlapping binding sites between cells prior to and after H_2_O_2_ stress (e.g., Atf1 and Pcr1) or between two repeats under the same condition (e.g., Sty1). Common major Atf1/Pcr1 binding sites are overlapping major Atf1 and major Pcr1 binding sites.

### Expression microarray data analyses

All expression microarray data were LOWESS normalized [53]. Significance analysis of microarrays (SAM) [54] was applied for identification of differentially expressed genes between wild type cells after H_2_O_2_ treatment for various times (e.g., 10, 30, 60, and 120min) and those prior to H_2_O_2_ treatment (e.g., 0min). To ensure the change of gene expression levels was not random, differentially expressed genes have to show the significance difference (i.e., fold change >2 and FDR<0.05) in at least two consecutive time points between cells prior to and after H_2_O_2_ treatment. Atf1 or Pcr1-dependent expression-response would be disrupted in *atf1* 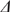 or *pcr1* 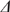 cells. To determine the Atf1 or Pcr1-dependent H_2_O_2_-responsive genes, we compared the (0min-normalized) transcriptional levels at various time points between *atf1* 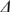 or *pcr1* 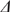 and wild type cells using SAM analysis. To ensure the non-random changes, response-disrupted genes in *atf1* 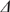 or *pcr1* 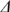 cells have to show the significant difference (i.e., fold change >2 and FDR<0.05) in at least two consecutive time points between *atf1* 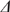 or *pcr1* 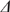 and wild type cells. The H_2_O_2_-responsive genes are listed in Table S5.

### Motif discovery scan

The 150bps sequences at the Atf1/Pcr1 binding sites were used in DNA-binding motif discovery. We applied Motif Discovery scan [44] methodology using the 30 top ranked Atf1/Pcr1 binding sequences to determine the binding motifs and using the next 30 top ranked Atf1/Pcr1 binding sequences to refine the motifs. The motif with the highest information content (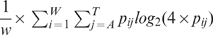
is presented. The receiver operating characteristic (ROC) curve of the motif was plotted by comparing the PSSM scores of the Atf1/Pcr1-bound promoter sequences (true discovery) and the unbound promoter sequences (false positive) to assess the genome-wide performance of the motif.

### Statistical analyses

Binomial test is applied to examine the significance of the non-random distribution, Fisher exact test is used to determine the significance of association between two groups, and *T*-test is applied to assess the significance of difference between two groups.

The supplemental materials File S1.pdf and Excel tables are available at the publisher website and the authors' website (http://pombe.gis.a-star.edu.sg/). The complete microarray datasets are available at the GEO database with the accession number GSE13053.

## Supporting Information

File S1 Supplemental information.
(0.88 MB PDF)

Table S1 List of Atf1 binding sites identified in cells prior to and after H_2_O_2_ treatment.
(0.08 MB XLS)

Table S2 List of Pcr1 binding sites identified in cells prior to and after H_2_O_2_ treatment.
(0.09 MB XLS)

Table S3 List of common major Atf1/Pcr1 binding sites.
(0.08 MB XLS)

Table S4 List of the Atf1/Pcr1-bound genes.
(0.06 MB XLS)

Table S5 List of the H_2_O_2_-induced and repressed genes.
(0.15 MB XLS)

Table S6 Phenotypic assessment of the H_2_O_2_-induced and/or Atf1/Pcr1-bound genes.
(0.03 MB XLS)

Table S7 List of genes whose promoter is bound by Sty1 in cells prior to H_2_O_2_ stress.
(0.14 MB XLS)

Table S8 List of genes whose promoter is bound by Sty1 in cells after H_2_O_2_ stress.
(0.08 MB XLS)

Table S9 List of genes whose intragenic sequences are bound by Atf1/Pcr1.
(0.03 MB XLS)

Table S10 List of the H_2_O_2_-induced Atf1/Pcr1 dependent genes.
(0.03 MB XLS)

Table S11 List of the H_2_O_2_-induced Atf1 or Pcr1 specific genes.
(0.03 MB XLS)
