# Supplementary material for: Genomic Binding Profiling of the Fission Yeast Stress-Activated MAPK Sty1 and the bZIP Transcriptional Activator Atf1 in Response to H2O2
Source: PLoS One. 2010 Jul 16;5(7):e11620. doi: 10.1371/journal.pone.0011620 (PMC2905393; doi:10.1371/journal.pone.0011620)
Supplement: File S1 — Supplemental information. (0.88 MB PDF) [file pone.0011620.s001.pdf]

SUPPLEMENTAL INFORMATION

Contents

Supplemental figures

Figure S1. Plating assay showing the phenotype of various strains used in ChIP-chip analysis

Figure S2. An overview showing the Atf1, Pcr1, and Sty1 binding profiles resulted from the ChIP-chip analysis

Figure S3. ChIP-chip enrichment of protein bindings at the Atf1/Pcr1-bound genes

Figure S4. Mini-culture growth curve assays for sensitivity to H_2O_2

Figure S5. Relationship between the Sty1 binding sites found in cells prior to and after H_2O_2 treatment

Figure S6. Relationship between two repeats of ChIP-chip experiment for Sty1 without H_2O_2 stress

Figure S7. Atf1/Pcr1-bound and unbound H_2O_2-induced genes show distinct dependencies of transcriptional response on Atf1 or Pcr1

Figure S8. Box-plot showing the Atf1 or Pct1 level changes at the induced or unresponsive genes

Supplemental figure legends

Figure S1. Plating assay showing the phenotype of various strains used in ChIP-chip analysis. Ten-fold serial diluted cultures of each strain indicated are spotted on YES plates supplemented with or without H_2O_2. Cells are grown on plates for 2-4 days at 30°C.

Figure S2. A chromosomal view showing the Atf1, Pcr1, and Sty1 binding profiles resulted from the ChIP-chip analysis. ChIP-chip result is visualized using SignalMAP software (NimbleGen, Roche). (A) An overview of the ChIP-chip analysis in chromosome III for various proteins under the growth conditions indicated. (B) A magnified view from (A) in the middle of chromosome III is shown.

Figure S3. Individual occupancies at the promoter of 158 Atf1/Pcr1-bound genes. Position of Atf1/Pcr1 binding sites (BS) is indicated at the bottom. A window of 2Kb in size at the binding sites is shown and separated by the vertical purple dash lines. Genes are shown on the top and the target genes are in red. Various protein occupancies in cells treated with (+) or without (-) H_2O_2 are shown in log2 scale. Gene names in each window from the left to right are ordered based on the gene number in Supplemental Table S4.

Figure S4. Mini-culture growth curve assays for sensitivity to H_2O_2. (A) Mini-culture growth curves of the 2-fold serial diluted cultures. The left panel showing that the half-maximal-concentration time T_{50} of each culture is proportional to the concentration of its starting culture. (B) The mini-culture growth curve of various strains in medium supplemented with 0, 1, or 2mM H_2O_2. The deficient score is defined by the ratio between ΔT_{50} in mutant and wild type strains.

Figure S5. Relationship between the major Sty1 binding sites found in cells prior to and after H_2O_2 treatment. (A) Venn diagram showing the relationship of major Sty1 binding sites identified in cells prior to and after H_2O_2 treatment. The intersection shows the overlapping major:major binding sites before and after H_2O_2 stress. Half or less of the major binding sites are found to overlap with the minor binding sites. Major binding sites that are not overlapped with minor ones are shown in parentheses. (B) Scatter plot showing the level of the Sty1 binding sites found in cells prior to and after H_2O_2 treatment. Correlation coefficient between the level of binding affinity before and after H_2O_2 stress is indicated. The overlapping major:major binding sites are shown in black; the remaining is in grey.

Figure S6. Relationship between the Sty1 binding sites in two repeats of H_2O_2 untreated cells. (A) Venn diagram showing the relationship of Sty1 binding sites identified in two repeats. (B) Scatter plot showing the level of Sty1 enrichment at various binding sites in two repeats.

Figure S7. Atf1/Pcr1-bound and unbound H_2O_2-induced genes show distinct dependencies of transcriptional response on Atf1 or Pcr1. Expression profiles of the H_2O_2-induced Atf1/Pcr1-dependent genes are separated into three groups: (A) the genes whose promoter contains major Atf1/Pcr1 binding sites (in red); (B) the genes whose promoter contains minor Atf1/Pcr1 binding sites (in grey); and (C) the genes whose promoter contains no Atf1/Pcr1 binding sites (in black). Circle, diamond, and triangle indicate the expression profiles in wild type, *atf1Δ*, and *pcr1Δ* cells, respectively.

Figure S8. Box-plot showing the change of Atf1 and Pcr1 enrichment levels in cells before and after H_2O_2 stress. Change of Atf1 and Pcr1 enrichment levels is measured by the ratio between the levels found in cells before and after H_2O_2 stress. Genes are grouped based on the H_2O_2 responsiveness. Ind-dep indicates the H_2O_2-induced Atf1/Pcr1-dependent genes; Unresp indicates the unresponsive genes, in which whose basal transcription is dependent on the Atf1 or Pcr1 are excluded.

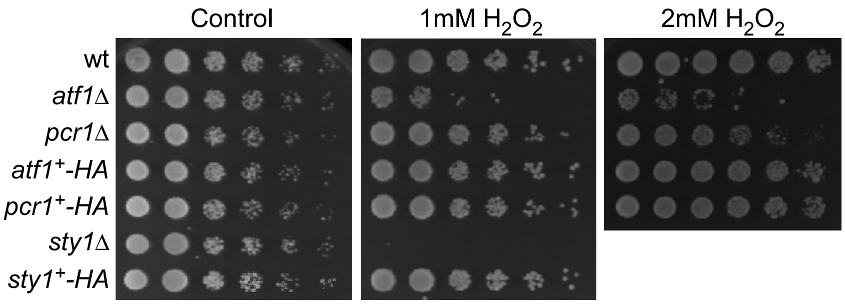

Sup Figure S1

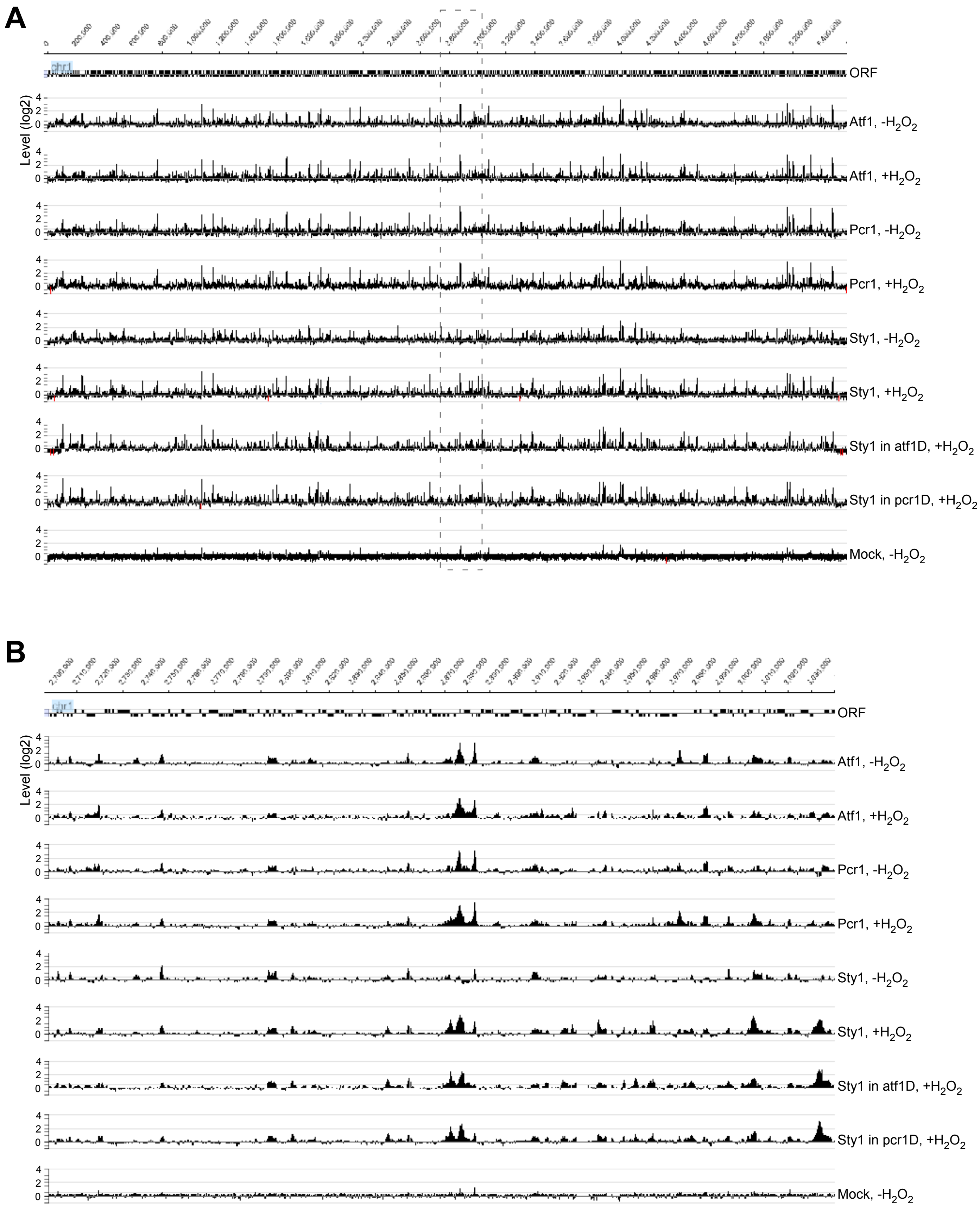

Sup Figure S2

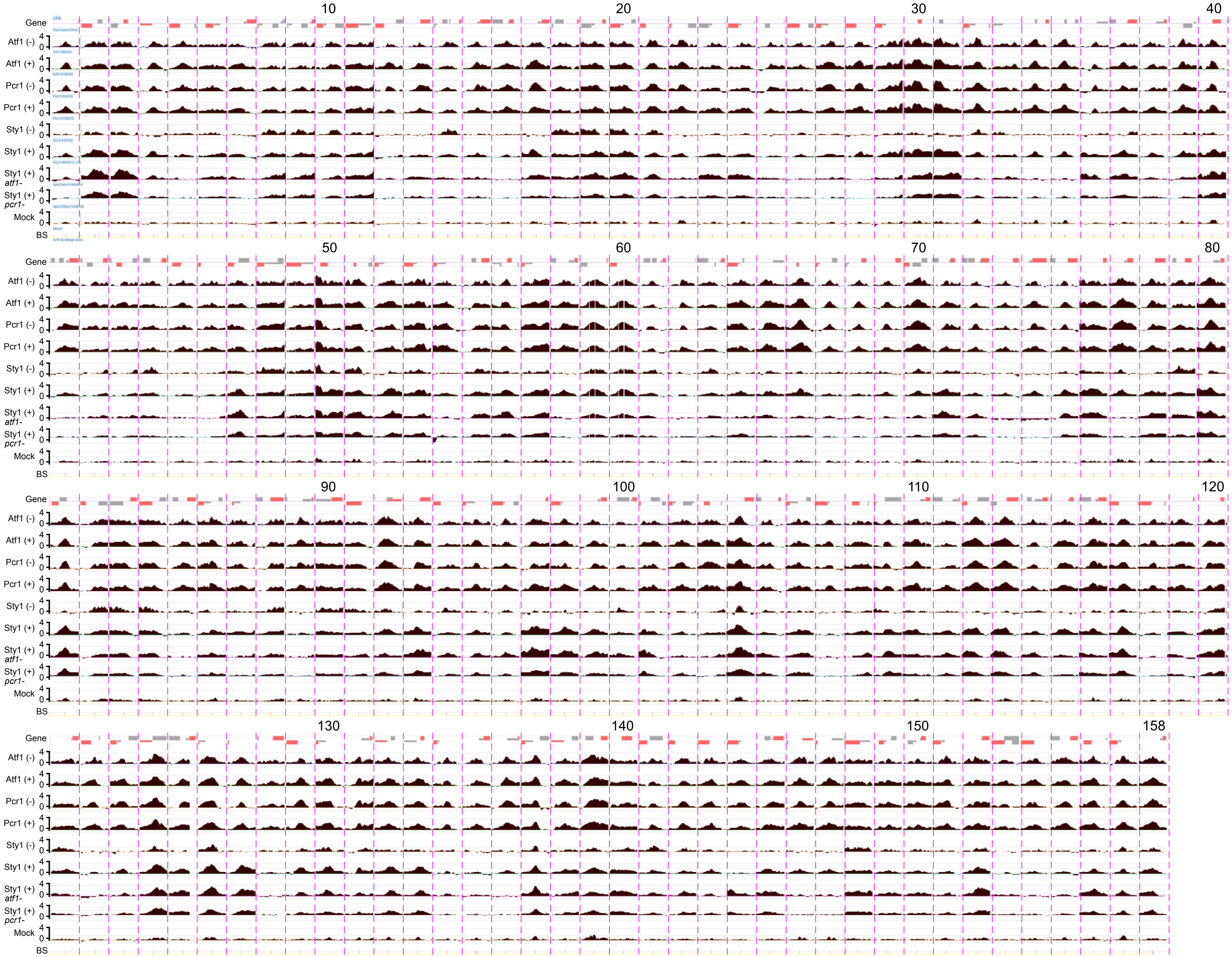

Supplemental Figure S3

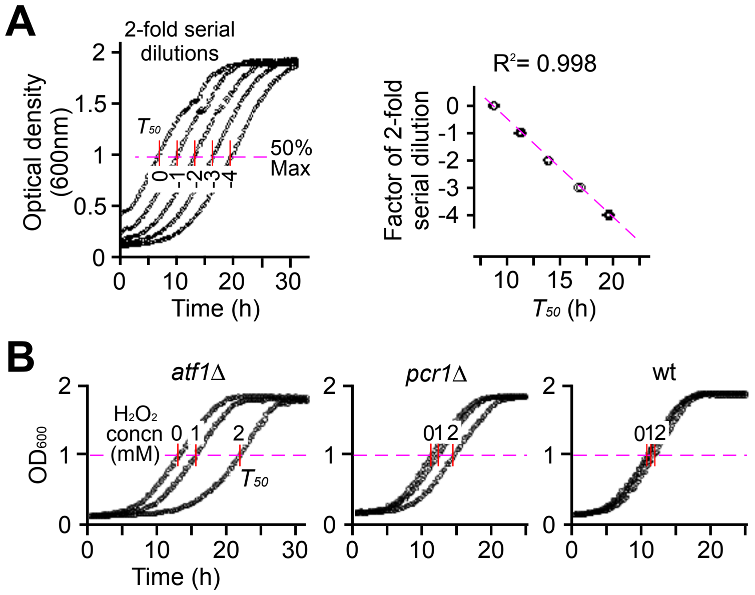

Sup Figure S4

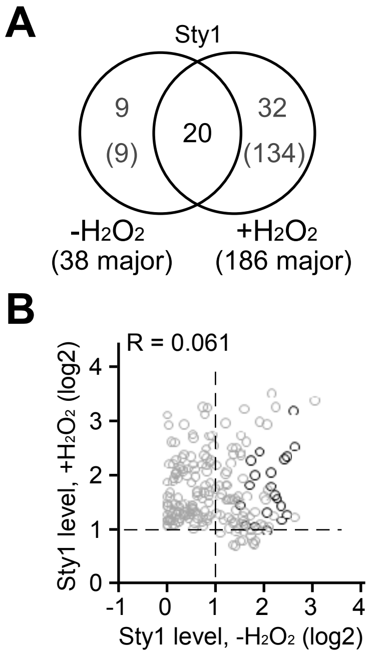

Sup Figure S5

A

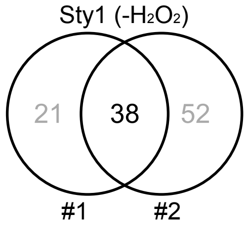

B

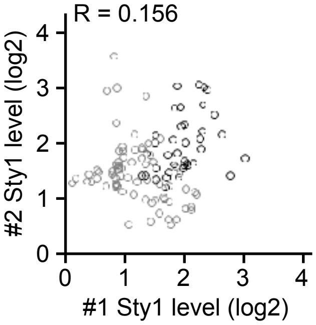

Sup Figure S6

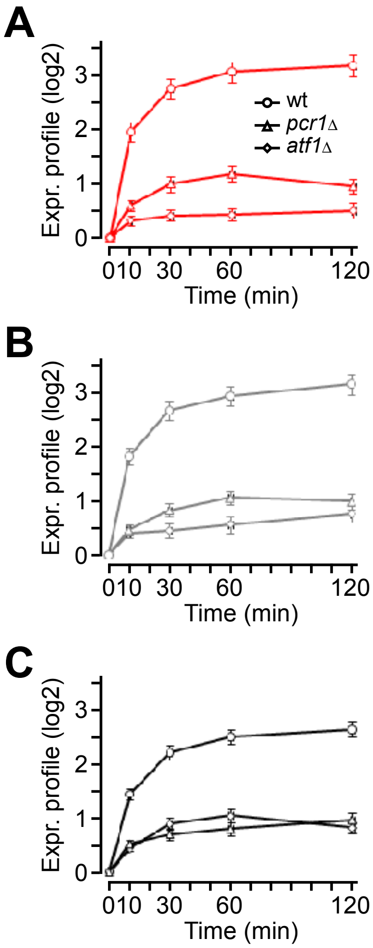

Sup Figure S7

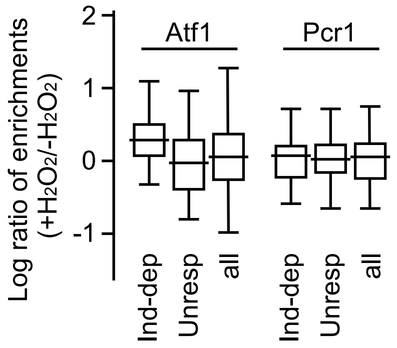

Sup Figure S8
